# History of the American Ambulance in Paris

**Published:** 1874-05

**Authors:** 


### Books Reviewed.

*History of the American Ambulance Established in Paris during the Seige of* 1870–71*; together with the details of its History and its Work. Vol. I.* By Thomas W. Evans, M. D., D.D.S., Ph. D., etc. London: Sampson Low, Manton, Low & Searle, 1873. New York: Wm. Wood & Co.

The book gives an interesting account of the labors of the American Ambulance in Paris during the Franco-Prussian War.

The first section of the work gives an extended account of the formation of the Sanitary Committee of Paris, and a history of the American Ambulance, during the seige of that city. This portion is from the pen of Dr. Thomas W. Evans. When war was first declared between France and Prussia, he conceived the idea of forming an Association for the purpose of putting into practical application, the improved method of treating sick and wounded soldiers, the utility of which the experience of the United States Sanitary Commission during the American Civil War, had fully demonstrated. Acting upon this idea he called a meeting of the American citizens resident in Paris, at which time a committee was appointed, consisting of Drs. Evans, Crane and Pratt, Col. Jas. McKaye and Mr. A. L. Ward, with power to add to their number, to act in connection with the "American Association for the relief of the misery of Battle-fields, "Société Internationale" and the Sociéte de Secours aux Blessés. Of this Committee, Dr. Evans was elected President and Dr. Edward A. Crane, Secretary.

The report of the organization of the American Ambulance, by Dr Crane, is divided into three parts, and comprises by far the largest section of the work.

Part I. considers the establishment of Army Hospitals, and is an extensive review of the whole subject. It takes into consideration all the devices which have been employed in time of war for the comfort and protection of the sick and wounded, and shows the mistakes which have been made in ill advised or poorly considered attempts to alleviate the miseries of the battlefield. From the experience of the past, he draws his deductions, and it for the purpose of putting these into practical application that the American Ambulance was established in Paris.

Part II. is a learned and somewhat lengthy article upon the use of tents and tents-barracks. The author treats of the subject from the earliest history of the employment of tents, down to the present time, and evinces a thorough knowledge of the subject. The article is valuable as a scientific inquiry into the use and relative value of tents, but the labor bestowed upon it seems to us hardly necessary in a work of this kind.

Article III. upon the special organization of the American Ambulance, complets this section of the work. It is a detailed account of the construction and conduct of the Ambulance, and is a valuable addition to the records of sanitary science. The surgical history of the Ambulance by Dr. John Swinburne, and a brief medical report by William E. Johnston, M. D., close the volume. These reports are full of interest and we cannot but deplore the brevity of the latter. That by Dr. Swinburue, gives a history of the Surgical treatment pursued in the Hospital, and reflects credit alike upon American Surgery and the organization of the hospital, which assisted materially in producing the results obtained. The appendix contains drawings of the Ambulance which will enable the reader to better understand the arrangement of the wards and the plans pursued in heating and ventilating them.

As the first volume of Dr. Evan's general history to be entitled "Sanitary Associations during the Franco-German War of 1870–71; this presents a fine appearance, and is a well written and valuable contribution to Sanitary and Medical Science. The press work is well done, and we congratulate Dr. Evans upon his production.

